# THE VALUE OF TECHNOLOGY IN LGBT OLDER ADULTS’ SOCIAL NETWORKS: LESSONS LEARNED FROM COVID-19

**DOI:** 10.1093/geroni/igad104.3705

**Published:** 2023-12-21

**Authors:** Kelseanne Breder, Walter Bockting, Elvan Ziyalan, Maureen George

**Affiliations:** New York University, New York City, New York, United States; Columbia University Mailman School of Public Health, New York City, New York, United States; Columbia University Mailman School of Public Health, New York City, New York, United States; Columbia University School of Nursing, New York City, New York, United States

## Abstract

LGBT older adults maintain unique social networks. They are more likely to live alone, more likely to rely on peers for caregiving, and less likely to have intergenerational support to adopt technologies that help maintain relationships. This research explores how LGBT older adults used technology for social support during COVID-19 social distancing and identifies recommendations for improving social isolation and wellbeing in this population through telehealth guidelines and community programs. Semi-structured individual interviews were conducted with a racially and ethnically diverse sample of 15 LGBT older adults during the summer of 2020. Interview guides were designed according to The Convoy Model of Social Relations. Verbatim transcripts were coded using conventional content analysis. Three major themes were identified, highlighting ways LGBT older adults used technology to meet social support needs, and the perceived gaps in technological fluency that hinder social connectedness and telehealth acquisition: (I) Yearning for “The Hug Factor,” (II) Navigating Online Social Boundaries and (III) “Not for My Generation.” Findings emphasize resiliency strategies employed by LGBT adults during social distancing and quarantine mandates that may optimize social and psychological wellbeing among the general aging population, as well as other minority geriatric groups. Our finding may hold relevance during future periods of isolation due to aging, mobility limitations, and climate change. Recommendations for improving social support beyond the pandemic include talk therapies, telehealth policy initiatives, and targeted community programs.

